# Targeting Network Imbalance in Schizophrenia: The Application of Temporal Interference Stimulation

**DOI:** 10.31083/AP50695

**Published:** 2026-06-25

**Authors:** Li Wan, Yiwen Chen, Nuo Chen, Qiong Ye, Shengchun Jin

**Affiliations:** ^1^Affiliated Psychological Hospital of Anhui Medical University, Anhui Medical University, Hefei Fourth People’s Hospital, 230022 Hefei, Anhui, China; ^2^School of Mental Health and Psychological Sciences, Anhui Medical University, 230032 Hefei, Anhui, China; ^3^Key Laboratory of Philosophy and Social Science of Anhui Province on Adolescent Mental Health and Crisis Intelligence Intervention, Hefei Normal University, 230601 Hefei, Anhui, China

**Keywords:** triple network model imbalance, default mode network, salience network, central executive network, temporal interference stimulation

## Abstract

Schizophrenia is a highly heterogeneous psychiatric disorder. Antipsychotics have limited efficacy on negative and cognitive symptoms, and are associated with side effects and high relapse rates. The core pathophysiology remains unclear, with most existing studies providing correlational neuroimaging descriptions lacking causal validation and precise intervention methods. The triple network model proposes that dynamic imbalance among the default mode network (DMN), salience network (SN), and central executive network (CEN) is a key feature of the disorder, underpinned by thalamocortical circuit disruption. Specifically, DMN hyperactivation, reduced SN connectivity, weakened CEN activation, and failure of SN-mediated network switching are observed. In schizophrenia, DMN hyperactivation initially serves as a compensatory response but eventually progresses to pathological decompensation. Conventional noninvasive stimulation techniques cannot effectively target deep brain nodes, limiting causal validation and precise intervention. As a novel noninvasive technique, temporal interference (TI) stimulation uses high-frequency alternating current interference to target deep brain regions, enabling both causal testing of network interactions and personalized modeling-based precise intervention. In summary, dynamic imbalance of the DMN-SN-CEN network is central to schizophrenia pathophysiology, with SN dysfunction as a potential causal factor. Targeting SN function using TI stimulation could directly test its causal role in network dynamics and symptoms, offering a route toward precision network intervention in this disorder.

## Main Points

1. Schizophrenia involves a dynamic imbalance among the default mode network (DMN) salience network (SN), and central executive network (CEN), not just local brain abnormalities.

2. SN dysfunction may causally disrupt DMN–CEN switching, leading to pathological brain states.

3. Temporal interference (TI) stimulation noninvasively targets deep network nodes, enabling causal testing and personalized intervention.

4. TI offers a path from correlational description to causal intervention in schizophrenia research.

## 1. Introduction

Schizophrenia is a highly heterogeneous psychiatric disorder characterized by hallucinations, delusions, emotional blunting, and cognitive impairments. While antipsychotic medications have proven effective for managing positive symptoms, they show limited efficacy for negative and cognitive symptoms. Side effects, high relapse rates, and treatment non-compliance further exacerbate long-term outcomes [1]. Only about one-third of patients achieve clinical remission, while another third experience chronic or progressive deterioration [2]. Non-pharmacological interventions, including transcranial magnetic stimulation and transcranial electrical stimulation, have demonstrated potential, but their effects are inconsistent and subject to individual variability [3,4,5]. This limited efficacy may stem from a crucial knowledge gap: the role of large-scale brain network imbalance and deep subcortical dysfunction in the disease remains poorly understood.

Among numerous theoretical frameworks, Menon et al. [6] proposed a triple-network model involving an imbalance among the default mode network (DMN), salience network (SN), and central executive network (CEN). This framework posits that the equilibrium among these three core networks is disrupted. The DMN, which includes hubs such as the medial prefrontal cortex and the angular gyrus, supports introspective processes such as autobiographical memory and must be suppressed during cognitive tasks. This suppression process is orchestrated by the SN. Centered on the anterior insula and dorsal anterior cingulate cortex, the SN is responsible for identifying behaviorally relevant stimuli and initiating a switch to the CEN. The CEN subsequently mobilizes attentional resources for working memory and goal-directed behavior. The thalamus projects broadly to all three networks, integrating and coordinating information flow. In schizophrenia, disruption of this thalamocortical circuit underlies the characteristic network imbalance, contributing to symptoms such as reality distortion or cognitive disorganization [7]. However, most research in this field remains confined to correlational neuroimaging findings, lacking methodologies capable of directly validating causal interactions among networks and thereby enabling the development of precise interventions. Recent advances in brain imaging, particularly resting-state functional resting-state functional magnetic resonance imaging (rs-fMRI), offer a window into these dysfunctions [8,9]. Simultaneously, noninvasive stimulation techniques like temporal interference (TI )provide a promising approach to modulate deep brain activity and validate causal network interactions [10].

## 2. Triangular Imbalance in the DMN-SN-CEN Network

The DMN is active during the resting state and suppressed during task states; however, in schizophrenia, it remains hyperactive even during cognitive tasks [11], contributing to symptoms such as hallucinations and delusions, and disrupting the perception of self and external reality [12]. The SN, which typically regulates attention and emotional conflict, exhibits significantly reduced connectivity in schizophrenia, impairing the ability to filter and prioritize relevant stimuli [13]. The CEN in schizophrenia shows reduced activation and weakened internal connectivity, leading to cognitive dysfunction [14]. In the healthy brain, the SN dynamically regulates the balance between the DMN and CEN, activating the CEN and suppressing the DMN during externally oriented tasks [15]; in schizophrenia, this switching mechanism fails, resulting in attentional rigidity and cognitive disorganization [9,16,17]. Emerging evidence suggests that DMN hyperactivation may initially emerge as a compensatory response to functional deficits in the SN and CEN, but may become maladaptive when this compensatory mechanism persists over time, ultimately trapping the system in a pathological state of imbalance [18]. We propose that the transition from compensation to decompensation represents a critical stage in the progression of network dysfunction in schizophrenia: DMN hyperactivity initially arises as a compensatory response to deficits in the prefrontal cortex (SN) and the CEN, but eventually culminates in a persistent state of imbalance.

## 3. Validating and Modulating the Triple Network Model

Current noninvasive stimulation techniques, such as transcranial magnetic stimulation (TMS) and transcranial direct current stimulation (tDCS), have difficulty delivering focused stimulation to deep brain network nodes, including the dorsal anterior cingulate cortex, anterior insula, and thalamus. This limitation hampers our ability to test causal interactions between networks and implement precise interventions in the human brain. The emergence of TI stimulation offers a solution to this bottleneck. TI stimulation delivers two high-frequency alternating currents (>1000 Hz) through scalp electrodes. At the intersection of the two electric fields, a low-frequency envelope (e.g., 5–40 Hz) is generated, which aligns with the intrinsic oscillatory rhythms of neurons, thereby modulating neuronal activity. Therefore, TI can noninvasively target deep subcortical structures.

Functional integration of the triple network is highly dependent on deep hubs located in subcortical and midline brain regions. For instance, the anterior cingulate cortex serves as a key node of the SN and a central relay station for interactions between the DMN and the CEN. The thalamus further acts as an integration gateway for multiple networks. TI generates a focused low-frequency modulation field in deep brain regions through the interference of two high-frequency alternating currents [19]. This approach therefore allows noninvasive, direct targeting of these deep nodes critical for network balance, including the dorsal anterior cingulate cortex (dACC) and anterior insula (AI, SN hub), the posterior cingulate cortex (PCC, DMN hub), and the thalamus. By modulating these nodes, researchers can now intervene in the core anatomical circuits, both the nodes themselves and their interconnecting pathways, that underlie dynamic interactions between the DMN, SN, and CEN.

Neuroimaging studies have revealed correlative abnormalities in network connectivity but cannot establish causal directionality. TI stimulation can serve as a tool for causal relationship verification. For example, by applying temporary, reversible inhibition to key SN nodes (e.g., dACC) via TI, researchers can simultaneously observe real-time changes in DMN and CEN activity. If inhibition of the SN exacerbates DMN hyperactivity and impairs task-related CEN activation, this would provide individual-level causal evidence that SN hypofunction leads to impaired DMN/CEN switching [20]. This targeted perturbation and whole-brain observation paradigm represents a pivotal step in advancing the triple network model from a descriptive theory to a causal science.

Neural oscillations mediate communication between networks. Different stimulation frequencies exert differential effects on network activity. While frequency-dependent effects (e.g., low-frequency inhibition, high-frequency excitation) are also achievable with repetitive transcranial magnetic stimulation (rTMS), TI’s distinctive advantage lies in its ability to deliver such frequency-specific modulation to deep brain structures that are inaccessible to conventional non-invasive techniques [21]. For a hyperactive DMN, low-frequency TI can be applied as an inhibitory intervention; for a hypoactive CEN, higher-frequency TI can be used as a facilitatory stimulation [22]. This frequency–function alignment allows TI to achieve both spatial specificity and physiologically meaningful modulation.

**Fig. 1.**
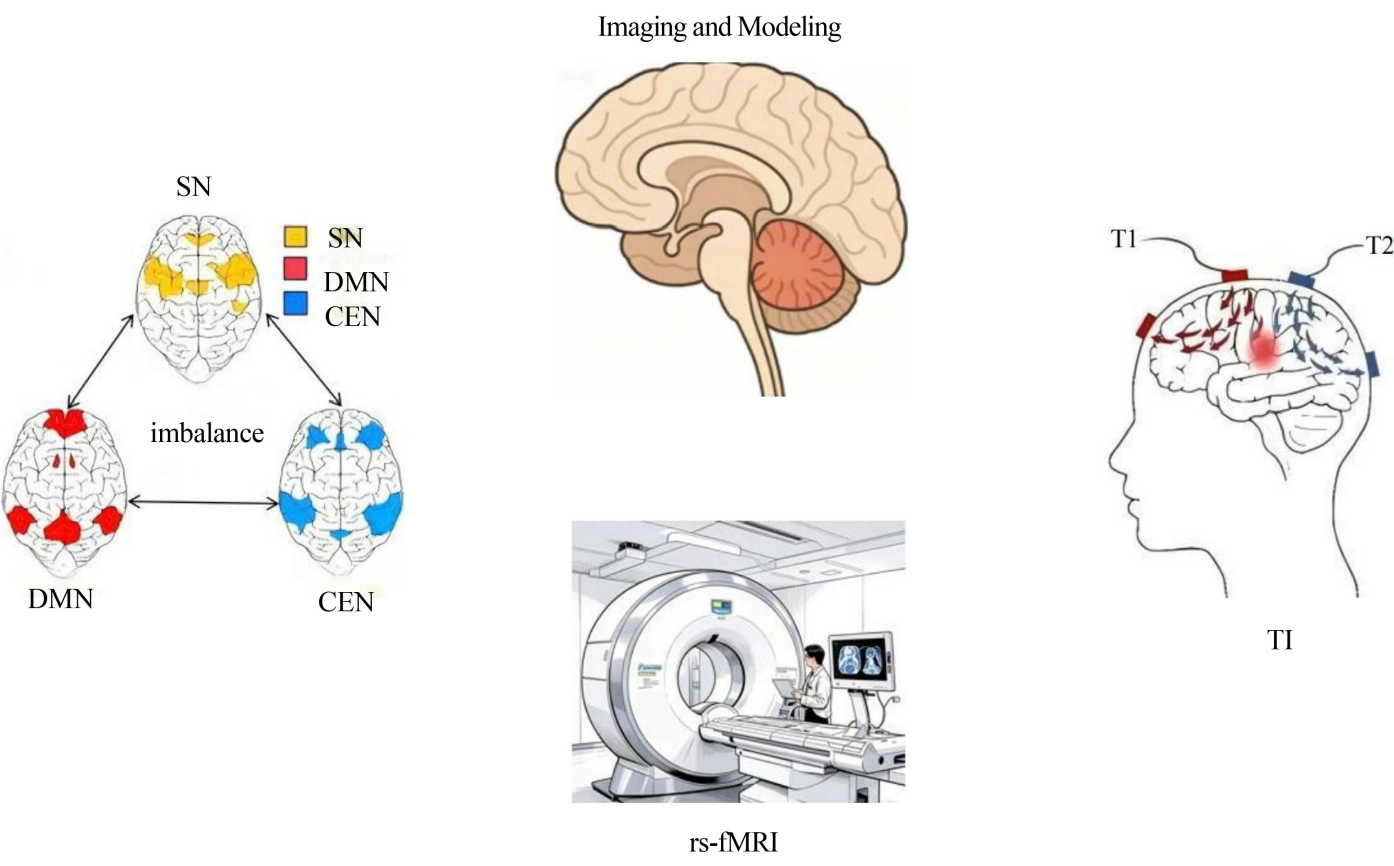
**Conceptual framework for investigating and modulating network dysregulation in schizophrenia**. SN, salience network; DMN, default mode network; CEN, central executive network; TI, temporal interference; rs-fMRI, resting-state functional magnetic resonance imaging.

Patterns of triple network imbalance exhibit substantial heterogeneity among patients. TI stimulation can be integrated with individualized computational models and task-based paradigms. Based on individual structural MRI data, finite element modeling can accurately simulate the intracranial distribution of the TI electric field. By combining these simulations with individual-specific network nodes identified via rs-fMRI, researchers can achieve personalized optimization of stimulation targets and parameters [23]. On the other hand, synchronizing TI stimulation with specific cognitive tasks (e.g., working memory, attentional switching) allows interventions to be delivered precisely when network functional demands are at their peak. This state-dependent stimulation holds the potential to break pathological network stability and guide brain networks toward dynamic equilibrium [24] (Fig. 1).

## 4. Future Directions and Specific Hypotheses

Building on the framework integrating TI stimulation and the triple network model, we propose a series of testable hypotheses aimed at translating theoretical constructs into research pathways and clinical interventions with well-defined causal chains. The core objective is to leverage TI’s causal probe property to directly test key pathological hypotheses of the triple network model. First, applying low-frequency TI inhibition to the dACC (a core node of the SN) during an attentional switching task in patients with schizophrenia is expected to significantly exacerbate DMN disinhibition and CEN hypofunction, accompanied by further deterioration in behavioral measures such as switching accuracy and reaction time. Such a finding would confirm that SN dysfunction is a causal factor in disrupting the dynamic balance between the DMN and CEN. Conversely, if applying low-frequency TI inhibition to the hyperactive posterior cingulate cortex (a core node of the DMN) at rest results in an immediate enhancement of functional connectivity between the SN and CEN, this would support the notion that DMN hyperactivity exerts an inhibitory influence on healthy network function.

To achieve precision medicine, interventions must be based on individualized maps of network imbalance, stratifying patients into neuroimaging-defined subtypes such as a DMN-dominant type or a CEN-hypofunction type, and delivering personalized TI stimulation to the relevant networks. We predict that this individualized strategy will lead to greater symptom improvement compared with control groups receiving sham stimulation or fixed-target protocols, such as conventional prefrontal cortex stimulation. Furthermore, sequential or simultaneous combined TI protocols are expected to produce synergistic effects on global cognitive function. These protocols involve low-frequency inhibition of the DMN paired with high-frequency enhancement of the CEN. Their therapeutic outcomes are expected to be superior to those achieved by single-network modulation. Further exploration of TI parameters and intervention paradigms may optimize treatment effects. The differential sensitivity of deep brain pathways to specific frequencies provides a basis for fine-grained neural regulation. Adjusting TI frequency at central nodes, such as the thalamus. For instance, using 5 Hz for inhibitory stimulation and 40 Hz for excitatory stimulation would yield distinct patterns of network modulation, thereby linking stimulation frequency to circuit-level responses and symptom changes. Moreover, implementing state-dependent TI stimulation during cognitive tasks involving the CEN, such as working memory training, may promote network reorganization and behavioral improvement more effectively than fixed-frequency stimulation delivered at rest.

### Limitations

Despite its unique advantages, TI stimulation faces several limitations that must be acknowledged. First, precise targeting requires individualized modeling based on structural MRI, which limits scalability. Second, optimizing electrode montages for deep focal interference without cortical spillover remains technically challenging. Third, empirical evidence on schizophrenia is scarce. Finally, direct validation of neuronal engagement in humans is lacking, with most evidence relying on computational models.

## 5. Conclusions

In summary, the core pathophysiology of schizophrenia can be understood as a dynamic imbalance among the default mode network, the salience network, and the central executive network, rather than as isolated local brain abnormalities. Within this framework, salience network dysfunction may represent a key causal factor that disrupts the switching mechanism between networks, thereby driving the brain into a pathological state of imbalance. As a technique capable of noninvasively targeting deep network nodes, TI offers a feasible tool for testing these causal hypotheses and implementing individualized interventions. Although challenges remain in terms of individualized modeling, electrode montage, and clinical evidence, TI integrates mechanistic inquiry with precise modulation, opening a new avenue for moving schizophrenia research toward causal intervention.

## Data Availability

This article does not generate new experimental data. All data supporting the conclusions in this article are derived from previously published references.
